# Protective Effects of Mesenchymal Stem Cells with *CXCR4* Up-Regulation in a Rat Renal Transplantation Model

**DOI:** 10.1371/journal.pone.0082949

**Published:** 2013-12-30

**Authors:** Zhiqiang Cao, Geng Zhang, Fuli Wang, Hongbao Liu, Long Liu, Yaling Han, Jian Zhang, Jianlin Yuan

**Affiliations:** 1 Department of Urinary Surgery, Xijing Hospital, Fourth Military Medical University, Xi'an, China; 2 Department of Nephrology, Xijing Hospital, Fourth Military Medical University, Xi'an, China; 3 Liaoning Province Translational Medicine Laboratory, Department of Urinary Urology and Cardiology of General Hospital of Shenyang Military Region, Shenyang, China; 4 State Key Laboratory of Cancer Biology, Department of Biochemistry and Molecular Biology, Fourth Military Medical University, Xi'an, China; University of Udine, Italy

## Abstract

The homing of mesenchymal stem cells to injured tissue, which is important for the correction of conditions such as ischemia-reperfusion injury (IRI) and immunolesions, has been performed previously, but with poor efficiency. Substantial improvements in engraftment are required to derive clinical benefits from MSC transplantation. Chemokines are the most important factors that control cellular migration. Stromal derived factor-1 (*SDF-1*) is up-regulated during tissue/organ ischemia damage, and its cognate receptor, chemokine receptor 4 (*CXCR4*), is involved in stem cell migration. The aim of our study was to investigate *CXCR4* expression in MSCs and to validate both its role in mediating migration to transplanted kidneys and its immunoregulatory effects in renal protection. Specifically, the present study was designed to investigate the short-term tissue homing of MSCs carrying genetically modified *CXCR4* in a rat renal transplantation model. We tested the hypothesis that MSCs with *CXCR4* over-expression can more efficiently regulate immunological reactions. Lentiviral vectors were used to over-express *CXCR4* or to introduce a short hairpin ribonucleic acid (shRNA) construct targeting endogenous *CXCR4* in rat MSCs. MSCs were labeled with enhanced green fluorescent protein (eGFP). After cell sorting, recipient kidneys were regionally perfused; recipient animals were injected with transduced MSCs, native MSCs, or PBS via tail vein following renal transplantation; and the effects of MSC injection were observed.

## Introduction

Mesenchymal stem cells (MSCs) have great potential for the treatment of various diseases, especially those involving tissue damage due to immune reactions and ischemia reperfusion injury (IRI) [1]–[3]. Acute/chronic renal failure, particularly renal allograft dysfunction, is associated with high morbidity and mortality [4]–[6]. An increasing number of studies have focused on endogenous and exogenous methods to protect renal function after renal transplantation [7], and MSC-based therapeutic approaches for organ transplantation are promising. Studies show that MSCs can prevent or attenuate ischemic tissue injury in primary transplantation [4], [8]–[11]. MSCs are specially characterized by their low immunogenicity and immunoregulatory abilities [12]–[17]. These MSC characteristics are ideal for their use in a renal transplantation model. Some studies have demonstrated that stem cells are capable of forming functional components of kidney [18], [19].

However, in vivo strategies with MSCs rely upon efficient localization and retention within the appropriate tissue(s). Current evidence suggests that in the absence of tissue damage, systemically administered MSCs only seed target tissues or organs at low levels [20]–[26]. Furthermore, due to localized hypoxia, oxidative stress and inflammation in the targeted tissue, the homing of transplanted cells is very also low and transient, reducing the therapeutic effects [26]–[28]. Thus, it is crucial to identify techniques that can enhance the chemotaxis and retention of implanted MSCs to maximize the effectiveness of MSC-based therapy. It is also important to elucidate MSC immunoregulatory mechanisms in transplanted kidneys. Many studies have demonstrated that stem cell migration and organ-specific homing are regulated by chemokines and their receptors [29], [30]. *SDF-1* plays a major role in the homing and engraftment of stem cells and progenitor cells to bone marrow and other injured tissues [23], [29], [31]–[33]. Its receptor, *CXCR4*, is highly expressed in MSCs within the bone marrow, but this expression is largely reduced during ex vivo expansion of MSCs [34]. Previous studies have shown that the *SDF-1*/*CXCR4* axis may play an important role in the homing and survival of MSCs [29], [33], [35]–[38]. However, the therapeutic effects of the *SDF-1*/*CXCR4* axis in renal transplantation have not been clearly evaluated, and its detailed mechanism of action is unknown.

The aim of our study was to modulate *CXCR4* expression in MSCs and to observe the effects both on secretory action and MSC viability in vitro and on migration and immunoregulation in transplanted kidneys in vivo. The surface expression of *CXCR4* was either up-regulated or knocked down in rat MSCs with lentiviral vectors. A rat renal transplantation model was utilized, and the homing, renal protection and paracrine/autocrine functions of these cells were assessed.

## Results

### Cells isolated from rat bone marrow samples exhibited the properties of MSCs

**Figure 1:**
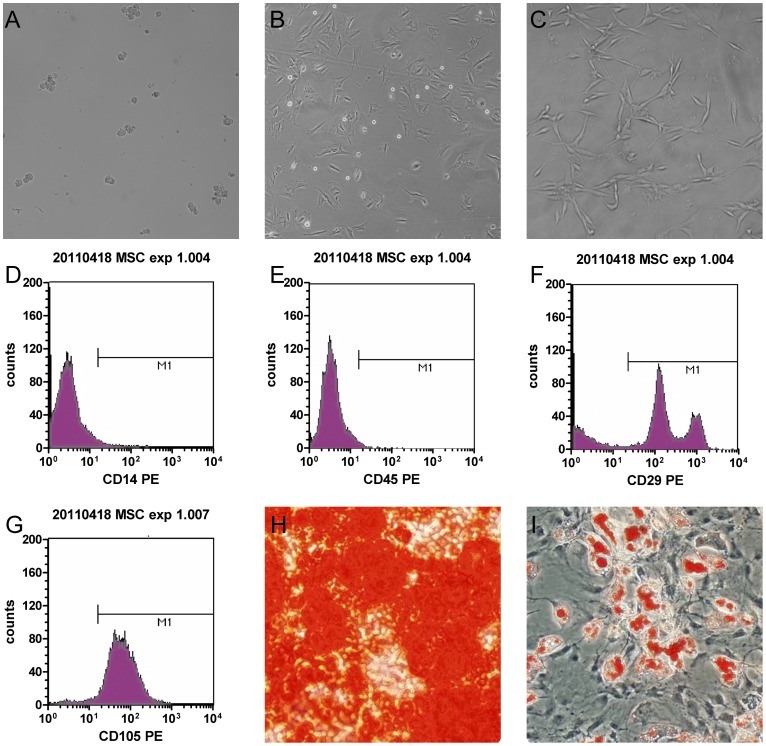
Characterization of rat bone marrow MSCs (α-MEM). (**A**) Isolated MSCs were round and small at 2 days after isolation. (**B**) After three passages, MSCs became larger and multiangular. (**C**) Before use, MSCs were mostly found in the clostridial form. (**D, E, F, G**) Fluorescent immunocytostaining showed that cultivated cells expressed CD29, strongly expressed CD105 and did not express CD14 and CD45. (**H**) Osteocyte lineage differentiation potential was determined by Alizarin Red S staining. (**I**) Oil Red O staining indicated the accumulation of oil droplets in cells that differentiated to adipocytes.

Primary adherent cells were small and round in the first few days following isolation (Fig. 1A), but later they became larger and polygonal (Fig. 1B). The cells were expanded under normal culture conditions and had a fusiform shape or uniform morphology after several passages (Fig. 1C). Rat MSCs expressed typical markers and differentiation profiles. They strongly expressed CD29 and CD105 but were negative for CD14 and CD45, as shown by flow cytometry analysis (Fig. 1D, E, F, and G). This surface marker pattern was comparable to previous studies and guidelines for MSCs [3], [39], [40]. Culture-expanded MSCs were also tested for their multi-lineage differentiation potential. In vitro tests using the appropriate inductive culture conditions promoted osteogenic or adipogenic MSC differentiation (Fig. 1H and I). Thus, the isolated cells met MSC standards mostly [40].

### Analysis of transfection efficiency

**Figure 2:**
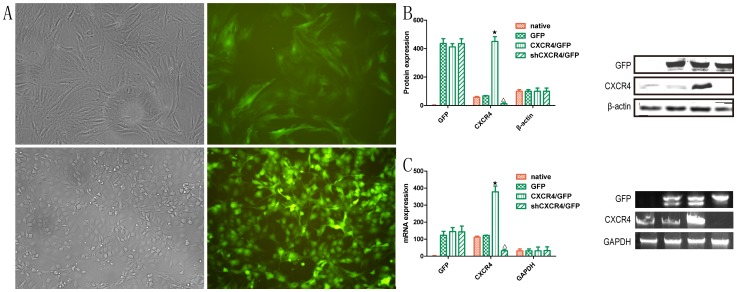
Transfection efficiency in MSCs. (**A**) Staining for fluorescence microscopy showed that MSCs expressed eGFP (original magnification 400× and 200×). (**B**) Western blot analysis was performed to detect *CXCR4* protein expression. β-actin was used as protein loading control. Analysis of *CXCR4* levels by histogram indicated that protein expression was up-regulated in MSCs*^CXCR4^* ^/GFP^(★P<0.05 vs MSCs^native^) and down-regulated in MSCs^sh*CXCR4*/GFP^ (▵P<0.05 vs. MSCs^native^). (**C**) Semi-quantitative RT-PCR was used to analyze *CXCR4* mRNA levels in MSCs. GAPDH was used as RNA loading control. (★▵P<0.05 vs. MSCs^native^).

MSCs were transfected with the sense-strand lentiviral vectors pLV-null-eGFP, pLV-shRNA-*CXCR4*-eGPF or pLV-*CXCR4*-eGFP. To confirm the transfection efficiency, MSCs were observed with fluorescence microscopy. Green fluorescent MSCs^GFP^, MSC*s^CXCR4^* ^/GFP^ and MSCs^sh*CXCR4*/GFP^ could be observed (Fig. 2A). The expression of *CXCR4* and GFP by MSCs was examined at both the mRNA and protein levels. Expression of the *CXCR4* protein was higher in MSCs*^CXCR4^* ^/GFP^ compared to cells from the control groups (MSCs^native^ and MSCs^GFP^) and MSCs^sh*CXCR4*/GFP^, as determined by Western blot (Fig. 2B). Semi-quantitative RT-PCR was performed and revealed that *CXCR4* expression was significantly higher in MSCs*^CXCR4^* ^/GFP^ than in the control groups and was lowest in MSCs^sh*CXCR4*/GFP^ (Fig. 2C). Furthermore, analysis of eGFP expression at the mRNA and protein levels confirmed the transfection efficiency.

### Effects of *CXCR4* expression on the cellular proliferation of MSCs

**Figure 3:**
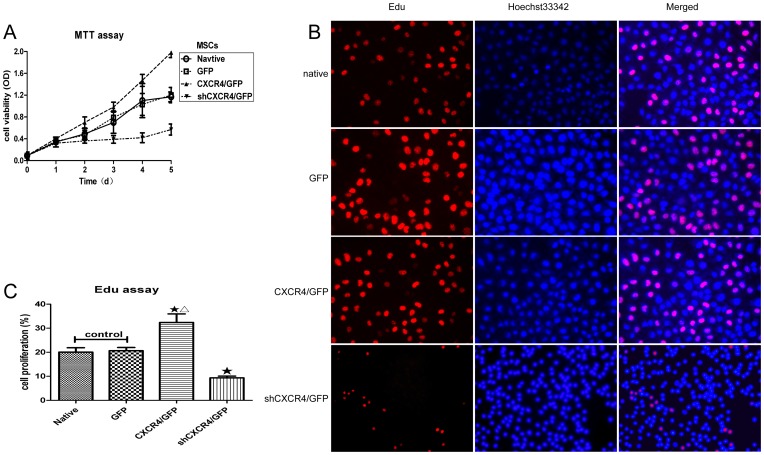
Regulation of *CXCR4* expression affects the proliferation of MSCs in vitro. (**A**) Viability was decreased in MSCs^sh*CXCR4*/GFP^ and increased in MSCs*^CXCR4^* ^/GFP^ compared to MSCs^GFP^ or MSCs^native^ (control), as measured by the MTT assay (Mean ± SD; n = 6; P<0.05 from NO. 4 to 5 time point). (**B**) All cell nuclei exhibited blue fluorescent Hoechst 33342 staining, and EdU labeling indicated replicating cells. In MSCs*^CXCR4^*, an increased number of cells exhibited red fluorescent EdU labeling following *CXCR4* overexpression. In MSCs^sh*CXCR4*/GFP^, fewer cells exhibited red fluorescence, indicating that EdU labeling decreased with *CXCR4* knockdown. (**C**) The percentages of red fluorescent cells for different MSC groups are indicated in the histogram. There were more replicating cells for MSCs*^CXCR4^* ^/GFP^ than the control group. ★P<0.05 vs. control, and ▵P<0.01 vs. MSCs^sh*CXCR4*/GFP^.

In order to investigate the proliferation and cytotoxicity, a standard proliferation and cytotoxicity test, the MTT assay, was adopted to assess mitochondrial viability, and a 5-ethynyl-2′-deoxyuridine (EdU) incorporation assay was used to investigate DNA synthesis in MSCs infected with either a *CXCR4* or shRNA-*CXCR4* lentiviral vector for *CXCR4* up-regulation or down-regulation, respectively. As shown in Fig. 3, knockdown of *CXCR4* resulted in significant reductions in MSC viability. (Fig. 3A). EdU incorporation dramatically increased from 28.2% to 42.3% in MSCs*^CXCR4^* ^/GFP^ (Fig. 3B) and decreased from 11.3% to 7.9% in MSCs^sh*CXCR4*/GFP^ (Fig. 3C). This result indicated that cell proliferation was inhibited with reduced *CXCR4* expression and that *CXCR4* up-regulation facilitated cell proliferation. Comparison of MSCs^GFP^ and MSCs^native^ showed no difference, demonstrating that the viral vector and eGFP expression did not affect cell proliferation.

### Effects of *CXCR4* regulation on MSC secretion

**Figure 4:**
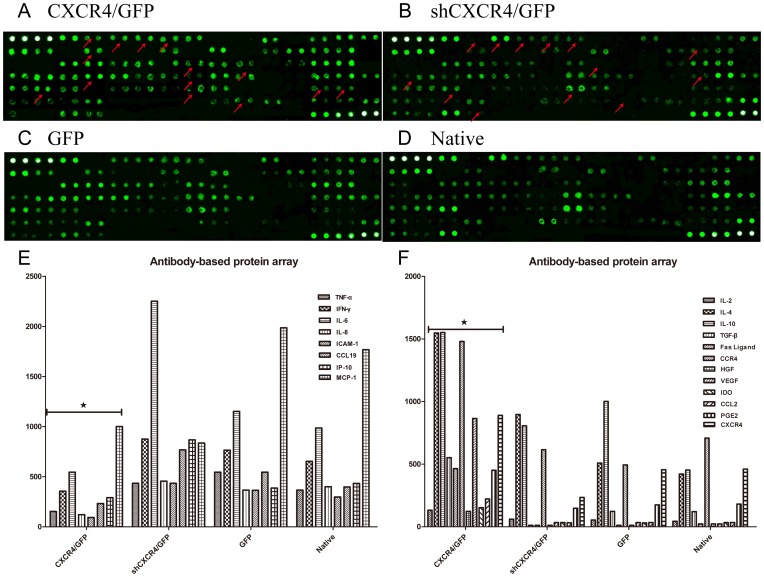
Antibody-based protein array system. Positive controls are located in the upper left-hand corner (four spots) and lower right-hand corner (two spots) of each membrane. (**A**) MSCs*^CXCR4^* ^/GFP^. (**B**) MSCs*^shCXCR4^* ^/GFP^. (**C**) MSCs^GFP^. (**D**) MSCs^native^. Down-regulated or up-regulated proteins are indicated with arrowheads. Histogram shows protein array results. (**E**) down-regulated proteins. (**F**) up-regulated proteins. ★P<0.05 vs. MSCs^GFP^.

We used a rat cytokine antibody array that enabled us to detect the expression of 90 different rat target proteins, including cytokines, chemokines, adipokines, growth factors, angiogenic factors, proteases, soluble receptors, soluble adhesion molecules and other proteins, in cell culture supernatants to investigate these factors in MSCs with and without modification. In contrast to MSCs^GFP^ and MSCs^sh*CXCR4*/GFP^, up-regulated factors in MSCs*^CXCR4^* ^/GFP^ included interleukins2, -4, -10 (IL-2, -4, -10), TGF-β, Fas Ligand, CCR4, HGF, VEGF, indoleamine 2,3-dioxygenase (IDO), CCL2, and prostaglandin E2 (PGE2), and down-regulated factors included TNF-α, IFN-γ, IL-6, IL-8, ICAM-1, CCL19, etc. (Fig. 4).

### *SDF-1* expression is up-regulated in transplanted kidneys

**Figure 5:**
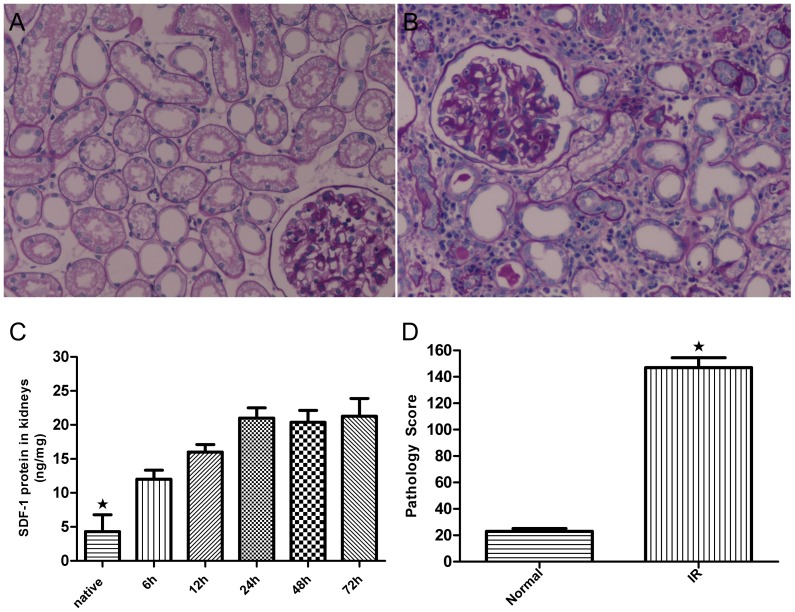
Injury in transplanted organs. (**A, B**) Morphology analysis showed apparent injury in transplanted organs. (**C**) *SDF-1* protein was up-regulated in transplanted kidneys. The kidney tissue lysates from recipient rats were analyzed by ELISA to determine *SDF-1* protein expression at 5 time points after transplant surgery. The histogram indicates that *SDF-1* expression was up-regulated in transplanted kidneys at different time points post-surgery time. ★P<0.05 vs. normal kidney. (**D**) Pathology score analysis showed a disparity. Normal kidneys were used as controls (methods not shown). ★P<0.05 vs. control.

In this study, we demonstrated that *SDF-1* was up-regulated in an IRI renal model (Fig. 5 A, B). The*SDF-1* protein levels in transplanted kidneys were observed by enzyme-linked immunosorbent assay (ELISA) at 6 h, 24 h, 48 h, and 72 h after surgery. The results showed that the IRI caused a time-dependent increase in *SDF-1* protein levels (Fig. 5C). *SDF-1* appeared to increase within the first 6 h after transplant surgery, peaked at 24 h and remained at high levels at 48 h until day 3 after surgery, compared to the levels observed in control kidneys. Pathology score analysis showed a apparently higher injury score in transplanted kidney (Fig. 5 D).

### Short-term homing of transplanted MSCs to transplanted kidneys *in vivo*

**Figure 6:**
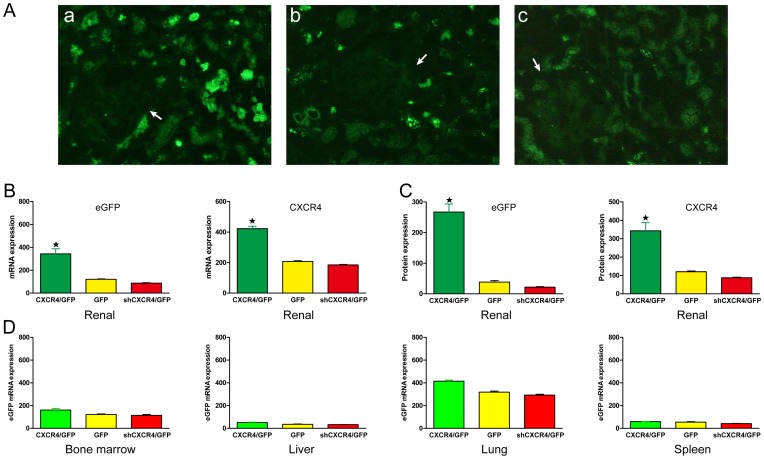
The role of the *SDF-1*-*CXCR4* pathway in the homing of MSCs. (**A**) Kidney uptake of eGFP-labeled MSCs was measured by fluorescence microscopy 48 h after systemic administration (a: MSCs*^CXCR4^* ^/GFP^, b: MSCs^GFP^, c: MSCs^sh*CXCR4*/GFP^). Labeled cells were detected surrounding the glomerulus (arrows). (**B and C**) Chemotaxis into transplanted kidneys was measured by RT-PCR andWestern blot. Recipients treated with MSCs^GFP^ were used as control. ★P<0.05 vs. control. (**D**) The expression of eGFP in different organs from recipient rats was measured 48 h after infusion.

To validate the homing of MSCs to target tissue, we looked for eGFP-labeled MSCs 48 h after MSC infusion. Transplanted MSCs could be detected via their eGFP expression by fluorescent microscopy. The migration and distribution of infected MSCs were observed 3 days after the operation. Many eGFP^+^ cells could be found in the transplanted kidneys (Fig. 6Aa, b, c). The vast majority of eGFP^+^ MSCs were located within the tubules. Some eGFP^+^ MSCs were also found around the lumens of blood vessels. Furthermore, *CXCR4* and eGFP expression were examined by quantifying mRNA and protein levels in the kidney via RT-PCR and Western blot, respectively (Fig. 6B and C). MSCs*^CXCR4^* ^/GFP^ demonstrated significantly higher levels of *CXCR4* and eGFP compared with MSCs^GFP^ and MSCs^sh*CXCR4*/GFP^.

Infused MSCs redistributed not only to the kidneys but also to other organs, including the lungs, spleen and bone marrow. We also detected eGFP expression by measuring mRNA levels via real-time fluorescent quantitation PCR in the lungs, spleen, liver and bone marrow of kidney recipients (Fig. 6D). It appears that *CXCR4* over-expression magnified the rate of homing to transplanted kidneys.

### Transplantation of MSCs ameliorate transplanted renal failure

**Figure 7:**
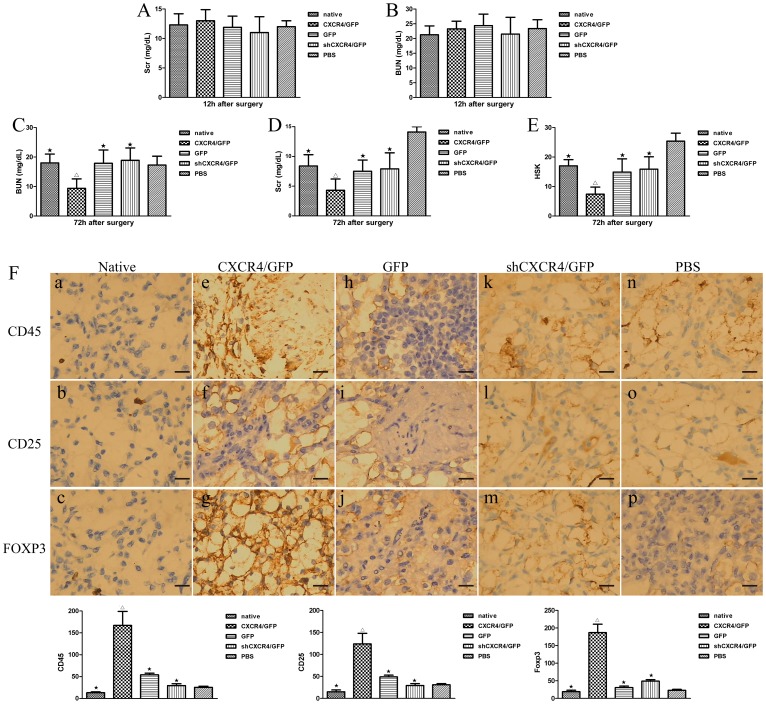
Transplantation of MSCs was renoprotective. The effects of the *SDF-1*-*CXCR4* pathway on the therapeutic efficacy of MSCs for the treatment of injury in transplanted kidneys was evaluated by measuring serum urea, serum creatinine, pathology scores, and immunohistochemisty staining. BUN and Scr levels were measured in recipients that received MSCs or PBS 12 h and 72 h after the surgery. There were no difference between each group 12 h after the surgery (**A and B**), but there were significant diversity 72 h after the surgery (**C and D**). ★P<0.05 vs. MSCs^CXCR4/GFP^; ▵P<0.05 vs. PBS. (**E**) HSK in transplanted kidneys that received different MSCs or PBS were calculated 72 h after the surgery (n = 8 per group). ★P<0.05 vs.MSCs*^CXCR4^* ^/GFP^ ; ▵P<0.05 vs. PBS. (**F**) Expression of CD25, FOXP3, or CD45 in the renal interstitium of native renal tissues or at different MSCs-treated groups after renal transplantation by immunohistochemistry. a, b, c: Paraffin-embedded sections of normal renal tissues showing weaker expression of CD25, or FOXP3, or CD45 [diaminobenzidine (DAB), original magnification 200×]. P<0.05 vs. transplanted kidney. d∼p: Paraffin-embedded sections of transplanted kidney exhibiting positive expression of CD25, or FOXP3, or CD45 in the renal interstitium (DAB, original magnification 200×). The expression of CD45,CD25 and FOXP3 were hyper in MSCs*^CXCR4^* ^/GFP^-treated group.★ P<0.05 vs. MSCs*^CXCR4^* ^/GFP^-treated group. ▵P<0.05 vs. PBS-treated group. Scale bars represent 50 µm.

Twelve hours after renal surgery, renal function was dissimilarly aggravated in animals receiving MSCs*^CXCR4^* ^/GFP^, MSCs^sh*CXCR4*/GFP^, MSCs^GFP^, MSCs^native^, and PBS treatment, as assessed by blood urea nitrogen (BUN) and serum creatinine (Scr) levels (Fig. 7A and B). The administration of all MSCs improved renal function assessed by Scr in animals at day 3 after transplantation compared to renal function in PBS-treated animals. However, MSCs*^CXCR4^* ^/GFP^-treated animals had significantly lower BUN and Scr levels at 48 h after infusion compared with MSCs^native^-, MSCs^GFP^-, and MSCs^sh*CXCR4*/GFP^-treated animals. Renal function was restored to normal levels at 3 days after transplantation in MSC-treated groups (Fig. 7C and D). BUN levels were also restored to normal levels at 3 days after transplantation in PBS-treated groups. But Scr levels did not. To further substantiate these results, histological scores of kidneys (HSK) were evaluated. As expected, kidneys from MSC-treated rats had significantly reduced HSK compared with control PBS-treated kidneys (Fig. 7E). Assessment of kidney function and structure showed that the transplantation of different MSC types, in particular MSCs*^CXCR4/^* ^GFP^, had greater therapeutic effects than the administration of PBS alone.

Immunohistochemisty staining showed that, within kidney interstitium, CD25 or Foxp3 immunoreactivity was detected in cells interspersed among the intertitium of renal tubules. In contrast with PBS-, or MSCs^sh*CXCR*4/GFP^-, or MSCs^GFP^-treated group, in MSCs*^CXCR4^* ^/GFP^-treated group, more percentage of CD25^+^ and Foxp3^+^ cells were present in the different zones of renal interstitium (Figures 7 F). But there were no different of CD45^+^ cells between the four groups (Figures 7 F e, h, k, n).

## Discussion

Renal transplantation is an effective approach for end-stage renal disease. To prevent rejection reactions, organ recipients must take steroids and immune-suppressing drugs for life after surgery. However, many serious side effects of these drugs, such as infection and tumors, impact recipient survival rates and quality of life. MSCs are characterized by their low immunogenicity, abundant tissue sources, easy accessibility, and immunoregulation abilities, and therefore, they have become a good option for organ transplantation. Professor Tan has achieved good therapeutic effects in clinical treatment [41].

This study investigated the effects of *CXCR4* expression modulation in MSCs on the role of MSCs in a renal transplantation model. The results showed that (i) the proportion of transplanted cells localizing into the kidneys increased with *CXCR4* overexpression; and the secretory action of MSCs was critically influenced by *CXCR4* gene modification.

Importantly, our observations confirmed a potential role for *CXCR4* in the short-term homing behavior of topically and systemically administered MSCs. We demonstrated that induced surface expression of *CXCR4* by lentiviral gene transfer was able to enhance in vivo short-term homing and to enhance cell proliferation and immunosuppressive soluble factors in vitro.

Site-directed administration of MSCs is only practical for a limited number of applications, and thus, the localization of MSCs is crucial for successful therapy. Current evidence suggests that in the absence of tissue damage, systemically administered MSCs only seed the target organ at low levels [21], [42], with large numbers of MSCs lodging in the pulmonary vascular bed [20]–[25], [43], [44]. The chemokine *SDF-1*, together with its receptor, *CXCR4*, plays a major role in the homing and engraftment of MSCs to target organs. Numerous studies have demonstrated that the *SDF-1*/*CXCR4* axis is essential for MSC homing in humans and rats [45], [46]. *CXCR4* and *SDF-1* levels are both up-regulated in stressed or injured tissues [11], [47]–[49]. Inflammation leads to activation of the endothelium, increased impermeability and the expression of various adhesion molecules. IRI-related acute inflammation causes acute organ damage and, more importantly, strengthens the host immune response by enhancing graft immunogenicity through activation of intragraft antigen-presenting cells and supporting infiltration by immune cells via up-regulation of major histocompatibility complex (MHC) class II (MHC II) antigens, intracellular adhesion molecule-1 (ICAM-1), and P- and E-selectin [50]. Therefore, IRI-induced intragraft inflammation is a key factor in renal allograft immunogenicity and explains poor outcomes [51]. MSCs, with their broad immunomodulatory and tissue-protective properties, might be ideal candidates for conditioning the environment in the recipient to combat IRI in transplanted organs [11], [49]. Reports on the homing and engraftment of MSCs are limited and controversial. Recent results showed that MSCs could migrate to damaged kidneys and participate in functional and structural recovery or regeneration [52]–[54]. However, subsequent studies have demonstrated that only a few MSCs engraft injured tubules, and their overall contribution to renal repair is negligible [26], [55]. Overall, in wild-type MSCs, *CXCR4* expression is limited [33]. MSCs can only home and engraft into IRI kidneys to a very restricted degree. Substantial improvements are necessary to enable greater clinical benefits. *CXCR4* expression is dynamic and can be regulated by cytokines, adhesion molecules, ligand-binding and proteolytic enzymes [56]. Previous studies demonstrated that such variability may be related to differences in culture conditions [30], [36], [57].

In the present study, we used a lentiviral system to stably over-express or knock down a functional *CXCR4* gene in rat MSCs, and we examined the effects in vivo. Based on Western blot and quantitative PCR data, as well as fluorescent microscopy, acceptable transgene expression levels were achieved. We did not determine whether other chemokines and their receptors also participated in the regulation of MSC homing in this study. However, we found that the neutralization of *CXCR4* could not completely abolish MSC homing and engraftment. This finding suggests that there are other factors that affect homing, which has also been suggested by other studies [58], [59]. Cooperative and compensatory mediators are involved in migration and homing, and these mediators can partially and flexibly substitute when *CXCR4* expression is lost. The cytokine milieu and the source of MSCs most likely both determine the main pathway that operates in cell migration [60]. Therefore, we examined changes to cytokines in this study.

We analyzed the cytokines present in MSC cultivation supernatant and determined that many interesting changes to protein levels occurred when *CXCR4* was modulated. Numerous in vitro studies have shown that MSCs have low immunogenicity [61] and poorly express or do not express HLA-I [13] or the transplantation immunity factors CD80,CD86,CD40,and CD40L [16]. In addition, MSCs have been shown to secrete cytokines and growth factors, which not only decrease IRI but also suppress immune cell functions [10], [17], [62]. MSCs inhibit the proliferation and cytotoxic effects of antigen-specific CTLs [63]. MSCs can also suppress T cell proliferation and activation [64]. In animal studies [65] and clinical trials [15], MSCs have been shown to exhibit immuno-depression and reduce inflammation. Their immunomodulation efficacy has also been shown in vivo during solid organ transplantation [41], [66]. However, the exact MSC immunomodulatory mode of action still remains unclear. Some reports have demonstrated that the immunomodulatory properties of MSCs are associated with direct cell-cell contact with T cells [67], whereas others have shown that MSCs can regulate immune functions by secreting a variety of cytokines and chemokines, such as interleukin IL-10, nitric oxide (NO), TGF-β, PGE2, HGF, and IDO [14], [17], [68]. These results are compatible with our study. The up-regulation of TGF-β and IL-10 is important for the differentiation and proliferation of regulatory T cells (Treg cells) [69]–[71]. Treg cells have very important immunoregulatory effects and play a significant role in the induction of immunotolerance or the maintenance of immunosuppressive activity [72]–[74]. Furthermore, up-regulation of many factors can enhance these immunoregulatory effects of MSCs, including CCL2 [75], PGE2 [76], [77],iNOS [78], VACM-1 and ICAM-1 [79], IL-4 [80], CD45 [81], and IDO [82], [83]. MSCs secrete soluble factors to regulate immunity. Our findings indicate the decreased immunogenicity of transplanted kidneys and the inhibited migration of activated T cells or possibly regulatory T cells that are necessary to mediate immunomodulatory functions [8]. It is well known that IFN-α induces MHC II expression and can also be amplified by TNF-α produced by other cells [84]. ICAM-1 expression could be affected by TNF-α and IFN-γ production, and it has also been reported that MSCs induce the down-regulation of ICAM-1 on co-cultured fibroblasts [85]. IL-2 and TGF-β can induce CD4^+^C25^−^ T cells to express Foxp3, converting them to CD4^+^CD25^+^ Treg cells [86]. Following the down-regulation of pro-inflammatory cytokines, both MHC II expression and subsequent injurious cell migration into injured kidneys might be suppressed by the administration of MSCs.

Another unique property of MSCs is their tissue repair potential, which is attributed to their migration and differentiation capacity and their ability to secrete various growth factors [21]–[23]. Although numerous IRI-related studies describe protective effects in kidney injury or transplantation models [9], [87]–[91], few groups have focused on the relationship between changes in the paracrine action of MSCs and the induction of immunotolerance. In our study, many factors relevant to immunosuppression were up-regulated when *CXCR4* was overexpressed.

Analysis of the effects of MSC application within an early time frame demonstrated *CXCR4*-related effects on three processes: (i) the down-regulation of pro-inflammatory cytokines (TNF-α, IFN-γ, IL-6); (ii) the inhibition of adhesion molecules (ICAM-1), resulting in diminished infiltration by macrophages and CD3+ T cells, particularly activated CD25+ cells; and (iii) the prevention of IRI-induced release of DC-attracting chemokines (CCL19), resulting in diminished infiltration by DCs. MSCs, through direct contact [92] or through the secretion of IL-6 or PEG2, inhibit the migration abilities of DCs, enhance the conversion of DCs into immature or tolerant cell types, or affect cell activity [68], [93]–[95]. Furthermore, we found that the over-expression of *CXCR4* could enhance these secretory actions. These data reveal a correlation between *CXCR4* expression in MSCs and intragraft immune activation that leads to acute rejection and compromised long-term graft function.

The secondary findings in our studies were the beneficial effects of *CXCR4* on cell proliferation and survival, which are very important in a therapeutic context. The longer that transplanted MSCs retain their special characteristics, the more transplanted MSCs can cycle and accumulate in ischemic kidneys, and the more recipient MSCs can be incorporated among renal cells, facilitating differentiation into renal stem cells or terminal cells and ultimately participating in the repair of kidney function. MSCs form nested capillaries that might be helpful in supplying more oxygen and providing secondary protection against injury tubular [96].

Some limitations of the current study are the relatively small sample size and a failure to study the ability of MSCs to induce tolerance and long-term graft survival in our model. In our next study, we will examine whether the up-regulation of *CXCR4* can also enhance the long-term residency of MSCs in transplanted kidneys. Several aspects of our study distinguish it from previous studies [97], [98]. These discrepancies may be explained by differences in experimental design, such as the cell transplantation protocol, the animal strain, and the type and severity of gene mismatch.

In conclusion, we have demonstrated that MSC therapy ameliorates the negative effects of IRI in a very strong, clinically relevant model of rat kidney transplantation at early time points. *CXCR4* plays a critical role not only in the process of homing but also in the pathogenesis of acute rejection and chronic allograft nephropathy, in which both immune- and non-immune-mediated mechanisms are involved. *CXCR4* is a clinically useful parameter for the identification of subjects with a high risk of acute rejection, chronic allograft nephropathy, and graft failure. The pretreatment to MSCs, such as using some cytokines or anoxia to up-regulate *CXCR4*, may facilitate the migration of infused MSCs to the site of injury and promote tissue repair [30]. Increased *CXCR4* expression can improve the homing of MSCs to transplanted kidneys, inhibit rejection reactions and accelerate the recovery of renal function in vivo. This simple method could contribute to the prevention of IRI and acute/chronic rejections and to the individualization of immunosuppressive therapies after renal transplant.

## Materials and Methods

All experimental procedures were conducted in accordance with the Detailed Rules for the Administration of Animal Experiments for Medical Research Purposes, issued by the Ministry of Health of China, and had received ethical approval by the Animal Experiment Administration Committee of the FMMU and the General Hospital of Shenyang Military Region (Shenyang, China). All efforts were made to minimize animal suffering and to reduce the number of animals used.

### Animals

Animals were purchased from the Animal Center of the Fourth Military Medical University (FMMU) (Xi'an, China) and the General Hospital of Shenyang Military Region (Shenyang, China), housed individually in cages with a 12 hour light-dark cycle and given free access to water and standard rat chow throughout the study. Eleven-week-old Wistar rats (200–220 g; Laboratory Animal Center of the FMMU, Xi'an, China) were used as donors, and 7-week-old Sprague-Dawley (SD) rats (200–250 g; Laboratory Animal Center of the FMMU, Xi'an, China) were used as recipients. Two-week-old SD rats (100–120 g; Laboratory Animal Center of the FMMU, Xi'an, China, and the General Hospital of Shenyang Military Region, Shenyang, China) were used as MSC donors. Animals were anesthetized with inhaled isoflurane (2% to 3%).

### Isolation of MSCs

Two-week-old SD rats were killed by cervical dislocation. Bone marrow cells from femurs and tibiae were collected using a syringe with a 26-gauge needle, and freshly isolated cells were centrifuged at 2,000 rpm for 5 min. The marrow was washed in phosphate-buffered saline (PBS), centrifuged at 1,000 rpm for 10 min, and then re-suspended into α-modified Eagle's medium (α-MEM; Gibco®, Life Tech, USA) supplemented with 10% fetal bovine serum (FBS, Invitrogen™,Life Tech, USA) and 1% penicillin-streptomycin (Gibco®, Life Tech, USA) in a humidified atmosphere of 5% CO_2_ at 37°C. After culture for 48 hours, non-adherent cells were removed via media replacement twice per week. When cultures reached 80–90% confluence, adherent cells were trypsinized (1% Trypsin–EDTA, Gibco®, Life Tech, USA). Cells were collected and replated at concentrations ranging between 0.05 and 0.15×10^5^ cells/ml of medium for several passages. MSCs from passages 4–8 were used for all experiments.

### Identification and differentiation of MSCs

To confirm the identity of the isolated cells as MSCs, the expression of some surface markers was examined by flow cytometry. Surface marker expression was measured by FACS analysis using specific mouse monoclonal antibodies for the rat surface markers CD14, CD45, CD29, and CD105, followed by staining with a PE-labeled anti-mouse-IgG specific antibody (all antibodies: BD Biosciences Pharmingen, USA).

The differentiation of rat MSCs (passage 4) into osteocytes and adipocytes was evaluated as described previously [3]. Briefly, to induce osteogenic differentiation, cells (6×10^3^/cm^2^) were seeded and cultured for 3 weeks in Dulbecco's Modified Eagle Medium (DMEM; Gibco®, Life Tech, USA) with 10% FBS and the osteogenic supplements 50 µM L-ascorbic acid-2-phosphate, 100 nM dexamethasone and 10 mM β-glycerophosphate (Sigma, USA). Bone mineralization was confirmed with Alizarin red staining. Adipocytes were identified by Oil red O staining (Sigma, USA) after treatment with induction medium, consisting of high-glucose DMEM with 10% FBS, 1 µM dexamethasone, 0.2 mM indomethacin, 0.5 mM 3-isobutyl-1-methylxanthine (Sigma, USA), and 10 µg/ml insulin (Novo Nordisk®), and maintenance medium, consisting of DMEM, FBS, antibiotics and insulin, for 3 weeks.

### MTT and EdU Assays

To determine whether *CXCR4* was involved in cellular proliferation, we infected MSCs with either a *CXCR4* or shRNA-*CXCR4* lentiviral vector for *CXCR4* up-regulation or down-regulation, respectively. The effects of genetic regulation on MSC proliferation were measured with a 3-(4,5-dimethylthiazol-2-yl)-2,5-diphenyl tetrazolium bromide assay and a 5-ethynyl-2′-deoxyuridine (EdU, Ruibo Biotech, China) incorporation assay using an MTT cell proliferation and cytotoxicity assay kit (Beyotime, China) and an EdU assay kit (RiboBio, China), respectively, according to the manufacturer instructions. OD values at 570 nm were measured with a Sunrise microplate reader (Tecan, Groedig, Austria). EdU-labeled cells were manually counted in ten fields of view that were randomly selected in each well, and percentages were calculated.

### Viral vector construction and transduction of MSCs

The lentiviral three-plasmid expression system (kindly provided by Prof. Jian Zhang, FMMU, Xi'an, China) was used to generate the recombinant vector. A rat *CXCR4* plasmid (kindly provided by Prof. ChaoJun Song, FMMU, Xi'an, China) was subcloned into the transfer vector pLV-IRES-GFP to generate the pLV-*CXCR4*-IRES-GFP plasmid, which contained the enhanced green fluorescence protein (eGFP) expression cassette, and insertion was confirmed by sequencing. Stable down-regulation of *CXCR4* was achieved by transduction of a lentiviral vector with short hairpin RNA (shRNA) for *CXCR4* (shRNA-*CXCR4*), generated by GenePharma (GenePharma, Shanghai, China), to knock down the expression of *CXCR4*. Complementary DNA oligonucleotides were then subcloned into the pLV-IRES-GFP backbone to generate the pLV-shRNA-*CXCR4*-IRES-GFP construct. This resulting transfer plasmid, a packaging plasmid (psPAX2), and an enveloping plasmid (pMD2.G) were co-transfected into 293T cells using Lipofectamine 2000 (Invitrogen™, Life Tech). The cells were transfected for 6 h, and the medium was subsequently replaced. The viral particles were harvested at 48 h or 72 h after transfection, filtered through a 0.45-µm cellulose acetate filter, and concentrated by centrifugation at 50,000 rpm (4°C) for 2 h. The titer was determined via transduction of 293T cells with serial dilutions of the vector and eGFP expression assessment by flow cytometry after 72–96 h. Infection with diluted pLV resulted in less than 40% eGFP^+^ cells, and this value was used to calculate the transducing units.

MSCs were transduced with pLV-IRES-GFP (encoding eGFP), pLV-*CXCR4*-IRES-GFP (encoding *CXCR4* and eGFP), or pLV-shRNA-*CXCR4*-IRES-GFP (encoding shRNA-*CXCR4* and eGFP, separated) lentiviral vectors at a multiplicity of infection of 30 in the presence of 5 mg/ml Polybrene (Sigma), followed by a second transduction after 48 h. MSCs transduced with pLV-*CXCR4*-IRES-GFP, pLV-shRNA-*CXCR4*-IRES-GFP, or pLV-IRES-GFP were referred to as MSCs*^CXCR4^* ^/GFP^, MSCs^sh*CXCR4*/GFP^, or MSCs^GFP^, respectively. In this study, MSCs^GFP^ and untransduced MSCs (MSCs^native^) were controls.

### RNA preparation and RT-PCR analysis

After humanely sacrificing the animals, organs were removed and kept in liquid nitrogen until analysis. Tissues were placed in lysis buffer and then homogenized with a tissue homogenizer using TRIZOL reagent (Invitrogen™,Life Tech, USA), and total RNA was isolated. Total RNA was isolated from MSCs using TRIZOL reagent as well. Reverse transcription-polymerase chain reaction (RT-PCR) was performed with equal amounts of RNA using a reverse transcriptase kit (Takara, Japan) according to the manufacturer's instructions. RT was performed in a 25 µl polymerase chain reaction reaction mixture that contained 10 nM 5′ and 3′ oligomers and Taq DNA polymerase (Takara, Japan). Real time-PCR experiments were performed using SYBR Green (Takara, Japan) and an ABI machine. Samples were normalized based on GAPDH values. The presence and levels of *CXCR4* or eGFP were determined with SYBR Premix Ex Taq kit (Takara, Japan) and a Rotor Gene 6000 Real-Time PCR Machine. The primers used for this study are available upon request. Gene sequences were searched in MEDLINE and re-validated. The temperature profile consisted of an initial step at 95°C for 10 min followed by 40 cycles of 95°C for 15 s and 60°C for 1 min. Melting curve analysis and agarose gel electrophoresis were performed after amplification. All of the results represent the average density of the positive bands obtained from three independent experiments using Quantity One software (Bio-Rad).

### Western-blot analysis

Kidney samples were homogenized, and the lysates were sonicated for 10 s and centrifuged at 12,000 rpm for 15 min. Protein concentrations were determined using a bicinchoninic acid (BCA) protein assay kit (Thermo Scientific). Fifty micrograms of protein was loaded onto a 10% SDS-polyacrylamide gel, and after electrophoresis, proteins were transferred to nitrocellulose filters. The filters were blocked with TBS-T buffer containing 5% nonfat milk and were then incubated with primary anti-*CXCR4* rabbit polyclonal antibodies (Santa Cruz, CA) or anti-eGFP rabbit polyclonal antibodies (Santa Cruz, CA). Equal loading of all lanes was confirmed by reprobing the membrane with anti-β-actin mouse monoclonal antibodies (Santa Cruz, CA) overnight at 4°C. Horseradish peroxidase (HRP)-conjugated secondary antibodies were obtained from Jackson ImmunoResearch Laboratories (West Grove, PA). Densitometric analysis was performed using Kodak Digital Science 1D software (Kodak, New Haven, CT). The experiments were repeated two more times with different tissues or pooled cells. The results were then statistically analyzed.

### Antibody-based protein array system

Cell-free supernatants were removed from the conditioned serum-free media of 4-day cultured MSCs^GFP^, MSCs^CXCR4/GFP^, MSCs^shCXCR4/GFP^, or MSCs^native^ and then analyzed with the RayBio Rat Cytokine Array V kit, which was purchased from RayBiotech (RayBio, Guangzhou, China). The array membranes can detect 90 different growth factors/cytokines at once. The layout of the membrane is depicted in Fig. 4. The assay protocol was followed precisely as stated in the directions from the manufacturer. In brief, each membrane was placed into the provided eight-well tray, 2 ml blocking buffer was added, and the membranes were incubated at room temperature for 30 min. The blocking buffer was decanted from each container, and the membranes were then incubated with 1 ml of conditioned medium at room temperature for 2 h. The samples were decanted from each container, and the membranes were washed three times with 2 ml of wash buffer I at room temperature with shaking for 5 min, followed by two washes with 2 ml of wash buffer II at room temperature with shaking for 5 min. One milliliter of 250-fold diluted biotin-conjugated antibodies was added to each membrane and incubated at room temperature for 2 h. After washing two times, 2 ml of 1000-fold diluted HRP-conjugated streptavidin was added to each membrane and incubated at room temperature for 30 min, followed again by 2 washes. The membranes were placed in detection buffer and incubated at room temperature for 5 min. Excess detection reagent was drained off, and the membrane was wrapped with PE wrap. The membrane was then exposed to Hyperfilm (Amersham Bioscience). Detectable spots were scanned by a densitometer. Positive controls, provided by the manufacturer, were normalized to 1-fold, and the densities of the unknown samples were calculated and normalized to the control spots.

### ELISA

The production of *SDF-1* in the kidney cortex was determined by ELISA using a commercially available ELISA kit (R&D Systems, USA) according to the manufacturer's recommendations. Tissue lysates were obtained by mincing, sonicating, and lysing with RIPA buffer. Protein content was quantified with the BCA protein assay (Thermo Scientific). All samples and standards were measured in duplicate.

### Animal model of kidney transplantation

After humane animal sacrifice, kidneys from male Wistar rats were harvested, perfused with Histidine Tryptophan Ketoglutarate solution (HTK, CUSTODIOL®, Germany) to remove blood from the vascular beds and maintained at 4°C. Approximatelyone hour later, kidney grafts were transplanted into male SD recipient rats, and blood flow was restored using standard microsurgical techniques. Contralateral kidneys were removed immediately after implantation of the left kidney graft. The animals were kept in a specific pathogen-free facility with drinking water containing Cyclosporine A (CsA, 1.5 mg/kg/day, Sandimmun Neoral; Novartis) and were injected with 1 ml PBS with or without 2.0×10^6^ MSCs via the tail vein 24 h after the operation (n = 8 per group). Amoxicillin (1 mg/ml) was given each day to prevent infection. Three days after transplantation, the rats were euthanized, and grafts and some original organs were harvested for Western blot, RT-PCR and histological analysis (n = 8 per group). Operated animals appeared healthy until just before graft harvest without surgical complications during the observation period.

### Cell transplantation procedures

To mimic the stem cell transplant protocol utilized in clinical renal transplant patients, SD recipient rats received lentiviral-transduced MSCs or native MSCs (2×10^6^) diluted in 1 ml PBS or 1 ml PBS alone as a control via straight perfusion and caudal vein injections. Straight perfusions were performed when draining blood from donor kidneys, and injections were performed 24 h after renal transplants.

Animals were randomly divided into five groups: the MSCs*^CXCR4^* ^/GFP^-treated group (n = 8), the MSCs^GFP^-treated control group (n = 8), the MSCs^sh*CXCR4*/GFP^-treated group (n = 8), the MSCs^native^-treated control group (n = 8), and the PBS-treated group (n = 8).

### Histopathological and biochemical analysis

To assess the therapeutic effects of native and genetically engineered MSC populations, blood was harvested 12 h and 72 h after renal transplantation, and tissue samples were harvested 72 h after transplantation. Biochemical and histopathological analyses were then performed (n = 8 per group). Briefly, random fields were analyzed using a 40× objective. The excised kidneys were fixed in phosphate-buffered 10% formalin, sectioned, and then stained with hematoxylin and eosin. HSK evaluation was performed in a blind manner by two separate pathologists. HSK was graded on a 4-point scale [99]: 0 = normal histology; 1 = mild damage [less than one-third of nuclear loss (necrosis) per tubular cross section]; 2 = moderate damage [greater than one-third and less than two-thirds of tubular cross section showing nuclear loss (necrosis)]; and 3 = severe damage [greater than two-thirds of tubular cross section shows nuclear loss (necrosis)]. The total score per kidney section was calculated by adding all 10 scores with a maximum possible injury score of 30. Quantification of Scr in serum samples was performed via an enzymatic method on a Cobas Integra analyzer (Roche, Indianapolis, IN). BUN quantification was performed manually via the diacetyl monoxime method.

### Immunohistochemical staining

To assess CD25, FOXP3, and CD45 expression in transplanted kidneys of four groups and normal renal tissues (Paraffin-embedded sections), The Paraffin-embedded sections were fixed in freshly prepared 10% paraformaldehyde for 5 min. After blocking the endogenous peroxidase activity with 0.3% hydrogen peroxide in TBS for 15 min, the serial sections (5-µm thick) were immersed in horse serum diluted 1∶10 in TBS for 30 min to reduce nonspecific binding, and then were incubated with antibodies against CD25, or FOXP3, or CD45 (Abcam, diluted 1 : 100,United States) overnight at after washing in TBS. Next, the sections were incubated in biotinylated IgG for 30 min, and avidin-biotin-peroxidase complex for 30 min. After each step of the staining procedure, the sections were given three 5-min washes in TBS. Immunoreactivity (IR) was visualized using 1 mg/mL diaminobenzidine as chromogen and 0.01% hydrogen peroxide as substrate. The peroxidase reaction was stopped after 5 min with distilled water, and the sections were counter-stained with Toluidine blue, dehydrated, and then mounted with Entellan. Slides were evaluated under a light microscope (original magnification200×). For digital image analysis, the software Imagepro-Plus was used. Results were scored by two independent investigators as hadro-positive (+++), positive (++), weakly positive (+), heterogeneous (+−), or negative (−). The two scores were averaged.

### Fluorescence microscopy for analysis of eGFP-positive cells

To determine whether overexpression of *CXCR4* enhances the chemotaxis of MSCs into transplanted kidneys, the presence of eGFP was used to distinguish resident MSCs from injected MSCs. For the in vivo migration assay with donor MSCs, the kidneys of euthanized rats were removed 3 days after transplantation, as previously described, and cryosectioned into 6-µm sections. The sections were observed under a fluorescence microscope (Olympus) to identify eGFP^+^ MSCs. Ten random fields were analyzed using a 40× objective. The number of labeled MSCs per visual field was estimated by Image J software.

### Statistical analysis

The results were statistically analyzed using SigmaStat (SPSS) version 11.0 software. All of the values were expressed as the mean ± SD. A one-way analysis of variance (ANOVA) or paired t-test was used for multiple or two-group comparisons. All of the tests were two-tailed, and a p-value of <0.05 was considered statistically significant.
